# Science and Society, Part IV: Psychologist Paul Eelen “Not a Single Football Player will Believe that he is Determined by the Ball”

**DOI:** 10.5334/pb.452

**Published:** 2018-07-26

**Authors:** Paul Eelen, Diederik Vandendriessche, Yves Dejaegere

**Affiliations:** 1 KU Leuven, BE

**Keywords:** interview

## Abstract

This manuscript is part of a special issue to commemorate professor Paul Eelen, who passed away on August 21, 2016. Paul was a clinically oriented scientist, for whom learning principles (Pavlovian or operant) were more than salivary responses and lever presses. His expertise in learning psychology and his enthusiasm to translate this knowledge to clinical practice inspired many inside and outside academia. Several of his original writings were in the Dutch language. Instead of editing a special issue with contributions of colleagues and friends, we decided to translate a selection of his manuscripts to English to allow wide access to his original insights and opinions. Even though the manuscripts were written more than two decades ago, their content is surprisingly contemporary. The present manuscript presents a translation of an interview with Paul Eelen by the University of Leuven student magazine Veto.

First published as: *Wetenschap en maatschappij deel vijf: de psycholoog Paul Eelen. “Geen enkele voetballer zal geloven dat hij gedetermineerd is door de bal”*. Interview in Veto Magazine, April 3 2000, 26, pp. 8–9. Interviewed by: Diederik Vandendriessche and Yves Dejaegere.^1^

As a behavioural and learning psychologist, Paul Eelen applies methods of exact sciences to living material, that is, humans. This means he seems to bridge the traditional divide between exact sciences and the humanities better than anyone else. His research material, however, threatens to throw a wrench in the works because it challenges a not unimportant facilitiy – human freedom.

Paul Eelen: “I first attended the seminary to become a priest. The first university degree I thus obtained was in theology. This was during the fifties, when it was custom in many Flemish families that one of their school-going children would opt for priesthood. But at a certain point, I no longer wanted to do this and wanted to study psychology instead. This wasn’t just a negative choice however. I was drawn into psychology. Psychology also revolves around the questions that people have about themselves. I did purposefully choose experimental psychology to steer clear of the philosophical problem.”

“The bishop had asked that I would hold off on my decision to quit theology for a year. He allowed me to already start studying psychology during that year in the meantime. I didn’t however often attend classes that year because I had already made up my mind and because I didn’t want to attend class in my religious habit. That year was pretty hard to be honest, primarily because of the statistics course. You should be aware that I had barely added up two numbers in the eight previous years. Psychology was also hardly known as a field of study back then. People who took me on board when I was hitchhiking had no idea how to understand my disclosure that I was studying psychology. It was something that was associated with psychiatry.”

## Chaos

Veto: What about this study drove you to make this your life’s work then? Or did this calling only develop along the way?

Eelen: “For me, psychology revolves around understanding what determines human behaviour. In addition, I had a clinical interest: I wanted to use it to help people in need. I ended up in the field of learning psychology. This field also has a behavioural question at its centre – how can our behaviour be modified? Within learning psychology, I also included a clinical aspect by means of behaviour therapy. However, the course I’ve most enjoyed teaching these last few years is the history of psychology. This course goes back to the fundamental questions. For example, it became clear that psychology developed at a time when people realised that all our knowledge is acquired through our senses. Combined with the progress made at the physiological level, this is where the foundation of experimental psychology lies. Applied psychology only developed in a later phase.”

“For a long time, what was called psychic was identified with consciousness, with language processes. But this excluded understanding the psychological characteristics of babies and animals. This has importantly changed because of behaviourism, although eventually behaviourists have limited themselves too much to strictly observable behaviour. In recent years, however, people have again started asking how the psyche should be defined and that answer is often being sought in the study of processes in the brain. Some say this evolution may herald the end of psychology as a science because it will blend into neuroscience. I believe this is a bit of an exaggeration because psychology essentially focuses on stimuli in the environment and how you respond to them. One will never understand human behaviour just through the mere study of processes in the brain. It is like a computer’s hardware and software. Psychology is clearly focused on the software, not on the hardware. But obviously the software needs the hardware.”

**“It’s a shame really that there is so little scientific discussion in the psychology faculty.”**

Veto: *Psychology was truly the prototype for the division between mind and body that has thoroughly determined Western thinking*.

Eelen: “That duality continues to cause trouble. There was a swift reaction to experimental psychology, which tried to apply methods from exact sciences. This opposition came from a humanities angle. If you look at the study programme that students are offered, you will notice that both schools are in fact separately presented. This duality continues to run strongly among faculty staff. However, there is in a large difference between applying methods of exact sciences to non-living material and to living people: one cannot avoid approaching the research question differently because one is dealing with a human subject. However, the reasoning behind it can be identical in both cases: one ultimately conducts an experiment in order to challenge a particular hypothesis. Nevertheless, it is becoming increasingly obvious that experimental research will never by itself yield thorough knowledge because human behaviour is determined by so many complex factors. These can never really be identified through isolated experiments. But nothing prevents observations from being made in experiments.”

“A lot is heard about chaos theory these days. Scientists start to realize that even positive science cannot predict how certain phenomena will manifest themselves. Then it is typically added that psychology is totally incapable of doing this. The example of boiling water is subsequently brought up: not a single scientist can predict where the first bubble of air will reach the surface. Cigarette smoke is another example: nobody can predict how it will circle in the air. So, OK there is chaos and indeterminability, yet nobody will question that the smoke goes upwards. I believe that in psychology, however, we even don’t know yet that the smoke will move upwards. Consequently, we should not worry that we cannot predict things down to the smallest details. Predicting human behaviour in a sense is similar to predicting the weather. Maybe one day, it will become possible to make very specific and detailed predictions as a result of complicated mathematical models, but until then only rough predictions can be made, and this is *a fortiori* also true for human behaviour. For now, we can only predict general tendencies. On the one hand, this means that you believe that human behaviour is influenced by one’s environment; on the other, we cherish the illusion of freedom and do not like to think of ourselves as determined by our environment. This is one of the most fascinating aspects of psychology. Is freedom something that contradicts determination by one’s environment? We are evidently influenced by our environment, but as soon as attempts to influence us become clear we start displaying opposite behaviour. Yet nobody will deny that this environmental influence exists.”

## Salon

“Several years ago, supermarkets hired people to collect the trolleys left around. You might attribute this behaviour to the characteristics of the people themselves: they aren’t paying attention; they don’t feel a sense of responsibility to put the trolleys back. From the moment the coin (20 Belgian Francs) system was introduced, one could only spot a trolley left behind every now and then. People’s behaviour changed massively without being told that people should pay better attention and show more civic behaviour. Simply giving a different consequence to a certain type of behaviour can strongly alter this behaviour. We attribute too many things to the internal characteristics of a person and pay too little attention to the environmental conditions for that behaviour. Some psychological concepts actually explain very little. For instance, the observation that ‘someone is not motivated’ when this person is not completing things. It would be better to identify the relation between completing a task and the environment’s response to it.”

Veto: This division sometimes also appears to exist in the psychology discipline. Aren’t different departments trying to explain the same phenomenon from a particular angle?

Eelen: “I sometimes have the impression that the different research fields are indeed strongly growing apart. There is very little mutual contact indeed. To be honest, it’s a shame that there is so little scientific discussion in the psychology faculty. There used to be a society: once a month, people would gather in the philosophers’ salon to have a glass of wine together. One colleague would then discuss his research which was the perfect opportunity to meet each other in a setting that was quite different from that of the departmental councils. This meeting is no longer organized. Very good individual research groups exist today, but there is little discussion between them.”

“In my view, every science should always imply a certain level of reduction. One cannot study everything from all viewpoints. Science is reductionistic by definition. There is only once science for which we hardly accept it and that’s psychology. This is so because this science involves ourselves and we don’t like being understood from one particular angle.”

## Internet

Veto: *Our first guest, history professor Lamberts, also mentioned human freedom. He said that humans after all are not limited to a bundle of atoms and for that reason they are not easily fitted into scientific models*.

Eelen: “Human freedom should of course play a role, but what do we mean by freedom? I’m not a football fan but I like watching football on TV. What I find so fascinating about it is that the course of the game is largely determined as soon as the first ball is kicked. The person who receives the ball is absolutely free to kick the ball forward or backward, but once it has been kicked, this determines the further game. Not a single football player will believe that he is determined by the ball. He will always have the impression that he is free to move or not to move, to kick it far or not to kick it far. What then is freedom? Our behaviour is determined at some level, but at the same time you never have the impression that you are tied up to a puppeteer’s strings. Everything the footballer does, he does in absolute freedom. He can opt to stay in place, but he won’t. Freedom is realizing that you should not have behaved in a particular way while you did so anyway. You had options, but you made anyhow a particular choice in the meantime. I for instance think that an animal is also free to act, but an animal will not realise that it could just as well not have done something. We know that we could have behaved differently; an animal doesn’t know this.”

Veto: You used the example of a computer earlier to represent the human mind. The metaphor of the internet is also very popular today. Is psychology letting itself be led by other scientific techniques?

Eelen: “For a long time, the clock was the leading metaphor. This was followed by artificial intelligence and today the so-called connectionist approach of the internet is the dominant one. The advantage of the internet approach is that you are looking for the functions of different structures. This creates more interaction between for instance psychology and genetics. Nobody would doubt that the colour of our hair is genetically determined, but the genetic determination of our behaviour of course brings up a whole range of new questions. Again, we are caught here in the nature-nurture debate. I’m more interested in the second part. Our behaviour certainly has a genetic foundation, but a great degree of variation can nonetheless manifest itself.”

“The other sciences have not only eroded areas of psychology; they have also contributed to asking more psychological questions. Today, attempts are made to investigating the same things from different scientific angles. We after all know that our behaviour is influenced by an enormous range of factors. It would be pointless to ignore all the knowledge from neural sciences. Psychology will always remain a science that it is not so much interested in the fact that people have brains – something we will never deny – but in what we do with that brain.”

## Ashtray

Veto: *Something that is striking about psychology is its love-hate affair with one of its founding fathers, namely Sigmund Freud. Psycho-analysis has always been very dubious at the scientific level. Yet it keeps resurfacing in university courses*.

Eelen: “Freud has had an enormous influence. Nobody will deny that the emphasis on the unconscious marked a breakthrough. I struggle with certain aspects of the theory that Freud developed because psychoanalysis does not advance falsifiable statements as its starting point. That does not mean that we should not recognise that our behaviour relies for a large part on unconscious processes. The question of what our consciousness actually is, is difficult in psychology. How do humans select information? When I recognise an object – say, this ashtray in front of me for instance – I don’t have any access to the reasons why I recognise it. Every perceptual process by definition proceeds in an unconscious manner. But how can we then still become conscious of this object? For me, consciousness is a much greater problem than the unconscious.”

“Freud contributed many meaningful ideas and perspectives. But after I have read him, I am left with the impression: “OK, so what?” It should always be possible to translate a scientific model or theory into something that can be investigated in a systematic manner. I don’t have anything against Freud’s conceptual framework, but one should use these concepts in a hypothetical sense. Otherwise one runs the risk of engaging in something that is not scientifically grounded. Science, on the other hand, should not explain everything. When you enjoy a sunset, you’ll never fully understand why you find that enjoyable. And nor should you have to – just enjoy it! We primarily ask ourselves questions when we end up into problems. We thankfully don’t do this for all the things that we feel good about. You should not begin asking yourself what caused you to fall in love after all. That makes the beauty of it go away. But it is useful to ask where those negative feelings are coming from if you have a depression.”

## Drama

Veto: Depression brings us to another aspect of psychology – psychotherapy. On the one hand, research on therapeutic models is done in psychology; on the other, medicine also offers psychiatric training. What is the difference between both trainings?

Eelen: “We are experiencing a very peculiar situation in this respect because psychotherapy training is being offered at the faculty of psychology, but the profession of psychotherapist officially remains a medical profession. This creates tension on both sides. Psychiatrists in my view have much too limited a background in psychology, while psychologists are not sufficiently versed in the biological foundations underlying certain therapeutic approaches. This is not a problem as long as you know that both are complementary. However, that complementarity is forgotten when everyone starts fishing in the same pond. A ground for conflict between psychologists and psychiatrists is always possible today. The relations with psychiatry can be very good when psychiatrists again primarily concern themselves with the biological foundations of disorders. But good collaborations will never be possible when everybody occupies the same space, with one of the two – the psychiatrist – having a bigger say for structural/organisational reasons than the other – the psychologist. For example, a psychologist cannot be the head of a centre for mental health at the moment. This anomaly requires structural solutions.”

Veto: What often happens is that psychologists quit once they start practising. Depressions during internships frequently occur. Is there not enough practice in psychology training?

Eelen: “This is certainly partially true. Especially during the last few years, it has become problematic because we developed into one of the university’s biggest faculties. Ninety percent of graduates do not have the ambition to go into research; the clinical sector still attracts the most interest. This means it becomes quite a challenge to offer a serious training programme with the limited staff we have. A lot of students don’t even learn well enough how to have conversation with patients during their training. If they aren’t properly guided during their subsequent internship, they lose the ground under their feet. It is tragic when you realise in your fifth year of study that you can’t handle the thing that you studied for during five years”

Veto: Could it not be a solution to introduce the internship at an earlier stage in the training?

Eelen: “That could be a solution, but those fundamental skills do require a certain maturity. It is difficult to imagine after all that a 19-year-old would have to help a depressed, 45-year-old man. That is disadvantageous for both sides: either the student is overwhelmed, or the patient says: what a youngster is expected to help me! Such problems also manifest themselves in the profession of a general practitioner: this profession is also not limited to prescribing a pill here and there. Doctors have to be able to talk with their patients.”

## Spiders

Veto: You said yourself that psychology was hardly known when you were a student. Psychology is fully established today, something that for instance shows in the large number of students. How do you explain psychology’s popularity?

Eelen: “Some people believe that the selectivity of the medicine educational programme explains the boom in recent years. People who did not pass the entry exam choose a different training with a doctor’s profile. It is also partially explained by the fact that ever more people choose something that interests them. The social sciences as a whole have seen their student numbers increase. There is much less of a focus on the commercial value of diplomas.”

“Many students often also have the wrong idea about the psychology educational programme. For instance, year after year it is obvious that students don’t know that courses in statistics are quite important. And this is often an obstacle for people who didn’t have a lot of math during secondary school.”

Veto: You mentioned your background as a priest at the start of the interview. Don’t you experience a conflict between your scientific activities and your faith at times?

Eelen: “I find it very difficult to involve my faith in my scientific activities. I don’t think there is a conflict, but I do consider them to be two separate worlds. The deeper you go into psychology, the more questions you start to have about faith. You have to constantly force yourself to keep religious explanations out of range because that would not be scientific. That, in turn, means you run the risk of excluding the faith dimension.”

Veto: The aspiration of banishing all of humans’ pathological characteristics sometimes pops up in genetics, which we previously discussed with professor Cassiman. When is a person pathological in the eyes of a pathologist? Where does the divide between pathology and health lie?

Eelen: “An important criterion for me is the question of whether that person or their direct environment are suffering from it. Take the example of someone who is afraid of spiders – and there are a lot of people with such fear. You can consider this a pathology when this fear becomes so big that the person no longer dares to enter his own house. Suffering is an important criterion because we are all pathological at some level. We all have our problematic characteristics but that doesn’t mean it is always needed to start therapy.”

Veto: What do you want to have taught your students by the time they leave university?

Eelen: “I would in fact want students to be pushed to think more thoroughly. We are still living in an educational system that predominantly rewards reproduction. I believe we don’t sufficiently teach students to think things through. Learning to think is something you often do by questioning the very simple things. How one challenges the obviousness of something is the road that leads to wisdom. Our study programmes create the impression that today’s theories will always remain valid.”

